# Nux Vomica

**Published:** 1888-08

**Authors:** A. B. Eadie


### NUX VOMICA.

*Characteristics.*

1. Aggravation at three A. M.
2. Disposition irritable, choleric, impatient, malicious, and spiteful.
3. The symptoms are worse in the morning, made worse by motion, exertion, and exposure to cool air; amelior-ated by repose and warmth. (Contra Puls.)

*Special indications.*

During the convulsions of Nux, the mind is not affected. (Contra Stram.)

The convulsions are generally interrupted by periods of calm, from which, however, they are renewed by the least noise, a breath of air, or the lightest touch.

The reflex function of the spinal marrow is unquestionably affected by Nux, which should be always in mind in convulsions from reflex irritation. (Acon.)

Convulsions from emotions, anger with anxiety, fright, vehemence; from indigestion, preceded by constipation and jaundice; opisthotonus with consciousness; limbs numb and rigid.

Renewed by bright light, sudden jar, noise, or the least touch; followed by deep sleep; whole body stiffened, pulse and respiration imperceptible; in the intervals twitching of extremities; trembling, ending in spasms; frightful tetanic spasms from least touch, as feeling the pulse; spasms with tetanic rigidity of nearly all the muscles of the body, with momentary interruptions, during which the muscles relaxed; violent tetanus, head drawn backward, arms extended, fingers flexed, jaws firmly contracted, face livid, eyes open, fixed, protruded; trunk stiff, legs extended and widely separated, feet turned out, toes flexed; skin dry and warm; respirations loud. Convulsions last two minutes, with intermissions of five minutes; renewed by noise, touch, jar, etc.

*Generalities.*

Aversion to open air; sensation of anxiousness in the body; pungent biting pains; bleeding from inner parts; congestion of blood to single parts; pain, as if being bruised, in outer parts; burning pain of exterior or of inner parts; too great irritability; inclination to lie down; aversion to moving; great debility, with over-sensitiveness; pain, as if being paralyzed; when walking staggers, drags feet;

| | |
|---|---|
| Worse in cold, dry weather; | Better in wet weather; |
| " cold air; | " warm air; |
| " lying on the back; | |
| " lying on right side; | " lying on left side; |
| " on moving. | " on reposing. |

*Differentiation.*

| NUX. | ACONITE. |
|---|---|
| Heat, with aversion to uncover; | Heat, with inclination to uncover; |
| Prevalent right side; | Prevalent left side; |
| Worse in snowy air; | Worse from heat of sun; |
| Most frequently improved by bending diseased part backward (*e. g.*, Opisthotonus); | Most frequently aggravated by bending diseased part backward; |
| Worse on right side; | Better on right side; |
| Nasal secretion watery. | Nasal secretion thick. |

A. B. EADIE.

